# Histone H3 lysine 27 acetylation profile undergoes two global shifts in undernourished children and suggests altered one-carbon metabolism

**DOI:** 10.1186/s13148-021-01173-8

**Published:** 2021-09-26

**Authors:** Kristyna Kupkova, Savera J. Shetty, Rashidul Haque, William A. Petri, David T. Auble

**Affiliations:** 1 Department of Biochemistry and Molecular Genetics, University of Virginia Health System, Charlottesville, VA 22908 USA; 2 Center for Public Health Genomics, University of Virginia Health System, Charlottesville, VA 22908 USA; 3 Laboratory Sciences Division, International Centre for Diarrhoeal Disease Research, Dhaka, 1000 Bangladesh; 4 Division of Infectious Diseases and International Health, University of Virginia Health System, Charlottesville, VA 22908 USA

**Keywords:** Epigenetics, Undernutrition, Stunting, Histone acetylation, One-carbon metabolism

## Abstract

**Background:**

Stunting is a condition in which a child does not reach their full growth potential due to chronic undernutrition. It arises during the first 2 years of a child’s life and is associated with developmental deficiencies and life-long health problems. Current interventions provide some benefit, but new approaches to prevention and treatment grounded in a molecular understanding of stunting are needed. Epigenetic analyses are critical as they can provide insight into how signals from a poor environment lead to changes in cell function.

**Results:**

Here we profiled histone H3 acetylation on lysine 27 (H3K27ac) in peripheral blood mononuclear cells (PBMCs) of 18-week-old (*n* = 14) and 1-year-old children (*n* = 22) living in an urban slum in Dhaka, Bangladesh. We show that 18-week-old children destined to become stunted have elevated levels of H3K27ac overall, functional analysis of which indicates activation of the immune system and stress response pathways as a primary response to a poor environment with high pathogen load. Conversely, overt stunting at 1-year-of age is associated with globally reduced H3K27ac that is indicative of metabolic rewiring and downregulation of the immune system and DNA repair pathways that are likely secondary responses to chronic exposure to a poor environment with limited nutrients. Among processes altered in 1-year-old children, we identified one-carbon metabolism, the significance of which is supported by integrative analysis with results from histone H3 trimethylation on lysine 4 (H3K4me3). Together, these results suggest altered one-carbon metabolism in this population of stunted children.

**Conclusions:**

The epigenomes of stunted children undergo two global changes in H3K27ac within their first year of life, which are associated with probable initial hyperactive immune responses followed by reduced metabolic capacity. Limitation of one-carbon metabolites may play a key role in the development of stunting.

*Trial registration* ClinicalTrials.gov NCT01375647. Registered 17 June 2011, retrospectively registered, https://clinicaltrials.gov/ct2/show/NCT01375647.

**Supplementary Information:**

The online version contains supplementary material available at 10.1186/s13148-021-01173-8.

## Background

**Fig. 1:**
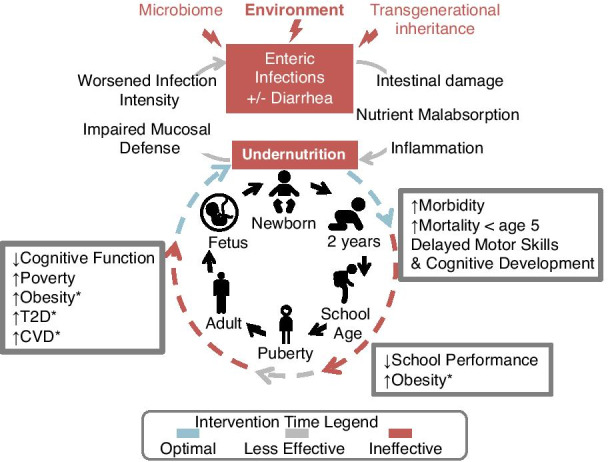
Vicious cycle of chronic undernutrition. Multiple factors contribute to chronic undernutrition in children. Enteric infections (with or without diarrhea) are key determinants of a child’s nutritional status, as they often lead to gut dysfunction with accompanying health issues listed in the figure. An optimal time for intervention is from conception until 2 years of age, a crucial time in child development [2]. A stunted child faces a high risk of life-long health consequences, which may be passed on to the next generation through poorly understood mechanisms. Health problems associated with the introduction of high calorie diet are marked with an asterisk. *T2D* type 2 diabetes, *CVD* cardiovascular diseases

Stunting is a global health problem in which a child does not reach their linear growth potential due to chronic or recurrent undernutrition. Stunting emerges within the first ~ 1000 days after conception, a crucial time in child development, and if left untreated it can lead to irreversible life-long health problems such as cognitive impairment, a dysfunctional immune system, vaccine failure, and significantly increased risk of mortality below the age of 5 years (Fig. 1) [1–7]. Introduction of a high-fat diet then leads to increased risks of type 2 diabetes, cardiovascular diseases and obesity [8–11]. Multiple factors contribute to stunting including nutrient limitation and lack of reliable clean water leading to increased exposure to pathogens. Pathogen exposure often induces episodes of diarrhea, resulting in histopathologic changes to the intestine and delayed or failed microbiome maturation [9, 12, 13]. Additional contributors to stunting include socioeconomic status, maternal health, and other environmental variables such as toxins [1–3]. In 2020, it was estimated that 21.3% (144 million) children under the age of 5 worldwide were stunted [5], with higher rates in low- and middle-income countries. The issue is so prominent that the World Health Organization (WHO) has set a goal to decrease the rate of stunting by 40% by 2025 [14]. To accomplish this, new nutritional interventions are needed, particularly those provided to children living in urban slum areas of low- and middle-income countries [7, 15].

We reasoned that the identification of epigenetic differences associated with stunted children would shed light on how the gene regulatory circuitry is modified in response to a poor environment. The epigenetic state of a cell is tightly linked to the availability of key metabolites [16], which makes it a fundamentally important area of focus in relationship to stunting. In our previous work, we showed that H3 trimethylation on lysine 4 (H3K4me3) profiles of stunted children undergo large-scale changes within the first year of life, which are similar to changes observed in cells grown in low methionine [17]. Here we expand on this finding by analyzing histone H3 acetylation on lysine 27 (H3K27ac). H3K27ac provides an indication of the relative activation state of transcriptional regulatory regions, particularly enhancers [18]. The discrete temporal changes in H3K27ac that we observe as stunting unfolds point to hyperactive immune responses in early infancy followed by metabolic downregulation by the time stunting is evident at 1 year of age. These patterns provide a framework for future development of biomarkers for at-risk infants and potentially new nutritional interventions to reset metabolic function, including one-carbon metabolism.

## Results

### Profiling the H3K27ac landscape in stunted and healthy infants

**Fig. 2:**
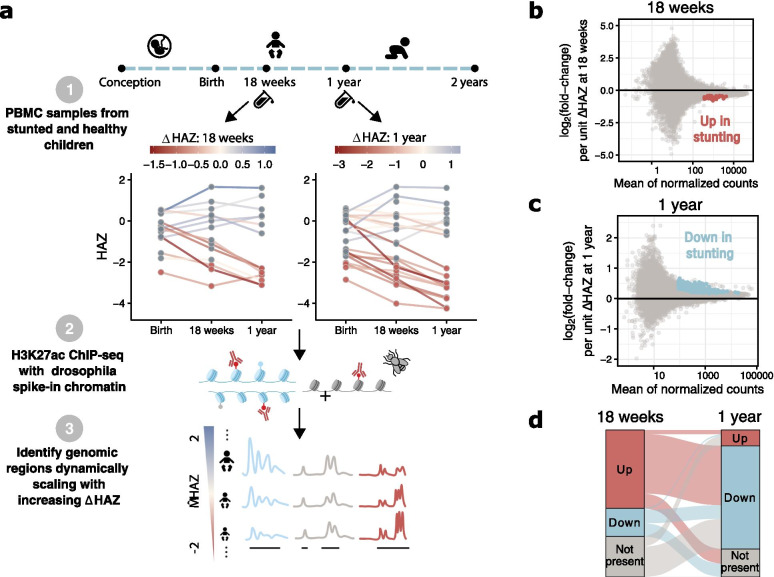
The H3K27ac landscape undergoes global changes in stunted children within their first year of life. **a** Overview of the experimental design. Each line in the plot with HAZ scores represents the change of HAZ score from birth to 1 year of age for a child whose PBMC sample was used for the study. The left panel shows HAZ scores for 18-week-old children (*n* = 14), the right panel for 1-year-old children (*n* = 22). Red dots indicate HAZ <  − 2 at a given age. Lines are colored based on ΔHAZ between birth and 18 weeks or between birth and 1 year. Bottom panel illustrates three different scenarios for differential analysis in which blue regions are downregulated in stunting, grey regions unchanged or with no significant trend; and red regions are upregulated in stunted children. **b** MA-plot shows changes in H3K27ac regions in 18-week-old children as the ΔHAZ (18 wk) score increases. Each dot is an H3K27ac region, the *x*-axis represents the mean read coverage over the region, and the y-axis indicates the log_2_(fold-change) of read coverage per unit increase of ΔHAZ (18 wk) score. Colored dots indicate significantly affected regions with false discovery rate (FDR) corrected *p* value < 0.05. **c** Same as **b** for 1-year-old children. **d** Alluvial plot showing changes to log_2_(fold-change) of corresponding regions between 18 weeks and 1 year. “Up”/“down”—H3K27ac levels are increased/decreased, accordingly, with higher risk of stunting at a given age, “not-present”—region was not identified at the given age

We obtained samples of peripheral blood mononuclear cells (PBMC) from 18-week-old (*n* = 14) and 1-year-old (*n* = 22) Bangladeshi infants enrolled in the PROVIDE (“performance of rotavirus and oral polio vaccines in developing countries”, total of 700 enrolled children) study [19] and performed chromatin immunoprecipitation followed by sequencing (ChIP-seq) to profile histone H3 acetylation on lysine 27 (H3K27ac) (Fig. 2a). We chose H3K27ac, a histone marks associated with active regulatory elements, as it has been shown to distinguish active from poised enhancers, and due to its roles in development [18, 20]. Stunting emerges within the first 2 years of life, and for this reason the first 2 years are considered an optimal time for therapeutic interventions. A child is considered stunted when their height-for-age *z*-score (HAZ) is below − 2 by 1 year of age. The available anthropometric data included each child’s HAZ scores at birth, 18 weeks, and 1 year (Fig. 2a, Additional file 1: Table S1), which allowed us to track epigenetic changes scaling with growth trajectory (the change in HAZ score over time, ∆HAZ), rather than simply using the phenotypic classification at a given point in time. In our prior work, we discovered that the analysis of epigenetic changes using growth trajectory afforded a much richer picture of chromatin changes than using phenotypic classification or HAZ scores alone [17]. The results obtained using ∆HAZ are more informative about factors associated with the development of stunting because the HAZ score often changes dynamically during early infancy, and children born with low HAZ scores are not certain to becoming stunted, and vice versa (Fig. 2a). Thus, the context provided by using ∆HAZ allows for more accurate and meaningful analysis.

The samples came from both male and female infants and with a broad range of HAZ and ΔHAZ scores (Additional file 2: Fig. S1a–c, j–l, Additional file 1: Table S1), and which are representative of the PROVIDE cohort overall (Additional file 2: Fig. S2a–e). Initial analyses did not suggest any differences in the number of H3K27ac enriched regions (peaks), putative enhancers, or superenhancers in association with any of the anthropometric *z*-scores (Additional file 2: Fig. S1d–i, m–r, Additional file 3: Table S2). We therefore moved on to analysis of H3K27ac levels in enhancer elements and their relationships with ΔHAZ scores.

### H3K27ac profile undergoes two global shifts in stunted children within the first year of life

We added *Drosophila melanogaster* chromatin as a spike-in control to determine if H3K27ac levels were globally altered among the samples (Fig. 2a, Additional file 2: Fig. S3, Additional file 3: Table S2). Principal component analysis (PCA) carried out on normalized H3K27ac counts shows a gradient change in the first two principal components (PCs) associated with ΔHAZ scores, where healthy children tend to locate in the upper right corner of the PCA plot and gradually progress to the lower left quadrant as the ΔHAZ score decreases (Additional file 2: Fig. S4a, b). The gradient is more apparent along PC1 and is especially pronounced in 18-week-old children. These results suggest that there are changes to H3K27ac levels associated with stunting in both 18-week-old and 1-year-old children.

Results of differential analysis versus ΔHAZ scores while controlling for sex indicated 38 significantly upregulated regions in 18-week-old stunted children (Fig. 2b, Additional file 4: Table S3), and 341 significantly downregulated regions in 1-year-old stunted children (Fig. 2c, Additional file 5: Table S4), the biological roles of which are discussed below. In addition to the significantly affected regions, we observed a significant global shift in H3K27ac indicating increased acetylation in 18-week-old stunted children (Fig. 2b, Additional file 2: Fig. S4c) and globally decreased acetylation at 1 year of age in stunted children (Fig. 2c, Additional file 2: Fig. S4d) compared to control children with higher ΔHAZ scores. This indicates that for children destined to become stunted, the H3K27ac landscape undergoes two major global shifts within the first year of life. Both shifts are statistically significant (*p* = 4.1 × 10^−196^, and *p* = 0 for 18-week-old children and 1-year-old children, respectively; Additional file 2: Fig. S4c, d). We also observed global shifts in the same directions in putative superenhancer regions, none of which, however, showed statistically significant association with ΔHAZ score (Additional file 2: Fig. S4e, f).

To test whether the observed patterns in the H3K27ac data might be driven by a specific subpopulation of PBMCs, we mapped the normalized H3K27ac signal onto cell type-specific enhancers defined by Andersson et al. [21], and calculated the average H3K27ac profile for each cell type (Additional file 2: Fig. S5a). If H3K27ac changes were attributable to a specific blood cell subtype, we would expect this particular subtype to show a pattern in which the average profile height would be positively or negatively correlated with the ∆HAZ scores. As shown in Additional file 2: Fig. S5b, c, the ordering of average profiles was similar across all the different cell types, suggesting that the shifts that we observe in stunted children are not indicative of changes in a specific population of blood cells compared to the others.

### Changes in H3K27ac between 18-weeks and 1-year of age

We matched enhancer regions from 18-week-old and 1-year-old children and compared the differential H3K27ac results directly to identify changes in enhancer acetylation that occur during this period of developmental time (Additional file 2: Fig. S6a). As a complementary strategy, we classified the differential acetylation results into three categories: (1) “up”—regions that were upregulated in stunting, (2) “down”—regions that were downregulated in stunting, and (3) “not present”—regions that were not detected at a given age (Fig. 2d). As expected based on the results that we have already presented, the largest proportion of regions were upregulated in 18-week-old stunted children and downregulated in 1-year-old stunted children (referred to as “up-down”; Fig. 2d, Additional file 2: Fig. S6a, b). The second largest group consists of regions that were not detected in 18-week-old children and were downregulated in 1-year-old stunted children (referred to as “not present-down”; Fig. 2d, Additional file 2: Fig. S6b). Interestingly, even though the “up-down” regions were the dominant group of changes between 18 weeks and 1 year, we did not observe any correlation between the changes associated with stunting at 18 weeks and 1 year of age (Additional file 2: Fig. S6a).

Next, we assessed the functional significance of regions in the two largest identified groups: (1) “up-down,” and (2) “not present-down,” by assigning the regions to their target genes followed by functional enrichment analysis of the associated genes. As shown in Additional file 2: Fig. S6c, clear patterns of pathway enrichment were observed. Genes with enhancers in the “up-down” category were associated with pathways involved in signaling processes, mRNA splicing, transcription-coupled nucleotide excision repair (TC-NER), transcription, translation, and the general stress response. These results suggest that infants destined to become stunted are not able to sustain responses to DNA damage and other stresses resulting from long-term exposure to a detrimental environment. Since TC-NER is activated by DNA damage in order to prevent cytotoxic transcriptional stress [22], TC-NER perturbations are related to deficits in transcription and associated pathways. The genes associated with “not present—down” regions show enrichment in immunological pathways, which suggests that the immune system of children with a high risk of stunting does not reach its full developmental potential.

### Gene targets of upregulated H3K27ac regions in 18-week-old stunted children

**Fig. 3:**
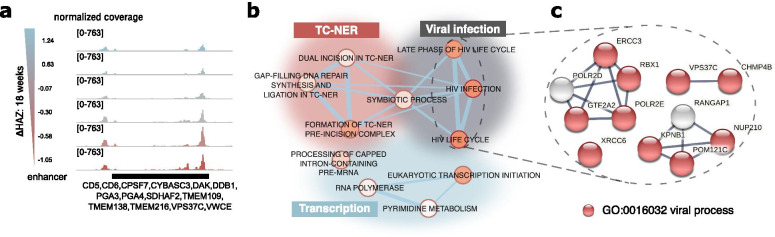
Functional annotation of genes associated with significantly upregulated H3K27ac regions in 18-week-old stunted children. **a** Genome browser snapshot of H3K27ac signal tracks showing a representative region in which the normalized coverage increased with decreasing ΔHAZ (18 wk) score, i.e. H3K27ac was higher in stunted children. The color of individual signal tracks corresponds to the ΔHAZ (18 wk) scores. Genes associated with the highlighted region are labeled. **b** Biological terms (databases: GO biological process, KEGG, Rectome, WikiPathways) that were significantly enriched for genes associated with differential H3K27ac regions in 18-week-old children. Each node is a biological term, the node size corresponds to the term size, the node color indicates the significance of enrichment (bright orange: low *q*-value, light orange: higher *q*-value, all *q*-values < 0.05), and the edges are based on the number of genes shared between terms. Groups of terms were manually organized in different colored clusters with the summary term for the cluster shown in the label with the same color. **c** Gene network constructed with STRING (see “Methods” section) using genes found in the highlighted biological terms in **b**. Red genes belong to the “viral processes” GO term

We next analyzed the 38 H3K27ac regions that showed significant association between H3K27ac levels and ΔHAZ (18 wk) scores (Fig. 2b). All of the regions show increased coverage with decreasing ΔHAZ (18 wk) score, indicating that H3K27ac levels progressively increased with the degree of stunting. An example region is shown in Fig. 3a. Functional annotation of target genes (Fig. 3b) identified pathways involved in TC-NER, transcription, and viral infection. Even though HIV infection pathways were seen to be enriched, further enrichment analysis of the genes found in these particular pathways showed that these genes are actually involved in general viral responses, as shown in Fig. 3c. These results suggest that children destined to become stunted face higher pathogen loads, possibly resulting from intestinal damage due to prior enteric infections [23], which additionally lead to increased DNA damage [24] among other associated problems. Eighteen-week-old infants may then activate the aforementioned pathways in response to these pathogen challenges.

### Functional assessment of differential H3K27ac regions in 18-week-old children

We next extended the exploration of possible cell subtype specificity described above to determine whether the differentially affected regions were particularly important for a specific cell type. The normalized chromatin accessibility signal from different cell types was mapped onto these regions, as shown in Additional file 2: Fig. S7a. The results in the top panel of Additional file 2: Fig. S7b show enrichment in blood specific open chromatin regions; however, there was no indication of a particular blood subpopulation driving the H3K27ac changes.

Next, we looked for enrichment of histone marks and DNA-binding factors within the significantly affected regions (Additional file 2: Fig. S8a, b, Additional file 6: Table S5, Additional file 7: Table S6). As expected, we saw the highest enrichment in H2K27ac (Additional file 2: Fig. S8a) followed by other activating histone marks. The only highly enriched repressive histone mark was mono-ubiquitinated histone H2A at lysine 119 (H2AK119) [25], which showed up only in stem cells. Interestingly, the mark with the second most significant enrichment was dimethylation of histone H3 on lysine 4 (H3K4me2), which has been previously shown to be associated with *cis*-regulatory regions driving cell-specific gene expression [26], and also with DNA repair [27]. This observation is consistent with the conclusion that stunted children have upregulated DNA repair pathways in early infancy. The top enriched DNA-binding factors (Additional file 2: Fig. S8b) mostly play roles in activation of T-cells and B-cells, as well as more general roles in cellular development.

### Gene targets of downregulated enhancer regions in stunting at 1 year of age

**Fig. 4:**
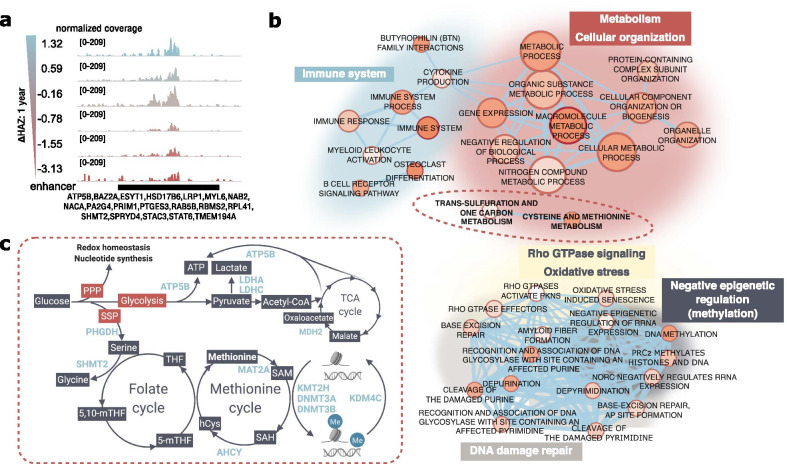
Functional annotation of genes associated with significantly downregulated H3K27ac regions in 1-year-old stunted children. **a** Genome browser screenshot showing a representative region in 1-year-old children in which read coverage decreased with decreasing ΔHAZ (1 year) score, i.e. H3K27ac was lower in stunted children. Track colors correspond to the child’s ΔHAZ (1 year) score. Genes associated with the region are labeled. **b** Network of biological terms as in Fig. 3b applied on data from 1-year-old children. **c** Genes from highlighted biological terms in **b** are shown in a pathway diagram. *PPP* pentose phosphate pathway, *SSP* serine synthesis pathway

To provide insight into epigenetic changes in 1-year-old stunted children, we performed detailed functional analyses of 341 regions with reduced H3K27ac levels in stunted compared to control children at 1 year of age. These regions were identified because of the positive correlation of H3K27ac with ΔHAZ (1 yr) score. An example of such a region is shown in Fig. 4a along with the list of its gene targets. Functional annotation of the set of target genes is presented in the network in Fig. 4b, in which we were able to identify functional submodules of pathways downregulated in stunting. Compared to the results from 18-week-old children suggesting an increased immune system response in stunting, stunting status at 1-year was associated with downregulated immune system pathways, suggesting possible immune system exhaustion, or “immunoparalysis” due to prolonged infections [6, 28], or problems in immune system development, as discussed above (Additional file 2: Fig. S6c). Similar to the immune system submodule, DNA damage repair pathways were predicted to be downregulated in 1-year-old stunted children in contrast to their upregulation in 18-week-old infants destined to become stunted. Submodules identified uniquely in data from 1-year-old children include negative epigenetic regulation (specifically methylation), Rho GTPase signaling, oxidative stress pathways, cellular organization, and metabolic pathways. Apart from general macromolecule metabolic processes, we were able to identify enrichment in one-carbon metabolic pathways, a simplified diagram of which is shown in Fig. 4c with target genes associated with downregulated H3K27ac regulatory regions listed in light blue.

### Region-centered analysis of downregulated H3K27ac regulatory regions in 1-year-old stunted children

As for the H3K27ac regions identified in 18-week-old children, we wondered if the significantly downregulated regions in 1-year-old children might be enriched in open chromatin regions of a specific cell type. The data shown in the bottom panel of Additional file 2: Fig. S7b indicates that these regions may be particularly important in CD14^+^ monocytes and CD1c^+^ dendritic cells.

Enrichment analysis for histone marks (Additional file 2: Fig. S8c, Additional file 8: Table S7) showed again that as expected, H3K27ac was a top hit, followed by mostly activating histone marks with the highest significance in monocytes and macrophages, as well as heterochromatin regions marked by trimethylated lysine 27 on histone H3 (H3K27me3) in T-cells and in B-cell precursors. The top hits in DNA-binding factor enrichment analysis (Additional file 2: Fig. S8d, Additional file 9: Table S8) include transcription factors driving immune responses to cytokines, growth factors, and interferons, again with the highest significance in monocytes. Interestingly, the enrichment analysis showed H3K4me2 (Additional file 2: Fig. S8c) and Jumonji Domain Containing 1C (JMJD1C) (Additional file 2: Fig. S8d) to be significantly enriched, both of which are associated with DNA damage response pathways [27, 29] and therefore provide further evidence that stunted children have altered responses to DNA damage.

### Integrative analysis of H3K27ac and H3K4me3 profiles in 1-year-old children

We next sought to integrate findings from this study with our previously reported results for H3K4me3 in stunting [17]. While there were no significant differences in H3K4me3 in 18-week-old children, the differences in H3K4me3 in stunted compared to control children were striking at 1 year of age. In contrast to the unidirectional changes in H3K27ac levels reported here (Fig. 2c), H3K4me3 changed in both directions in relationship to ΔHAZ (1 yr) (Additional file 2: Fig. S9a, Additional file 10: Table S9), such that H3K4me3 was redistributed from TSS-proximal sites to distal locations.

**Fig. 5:**
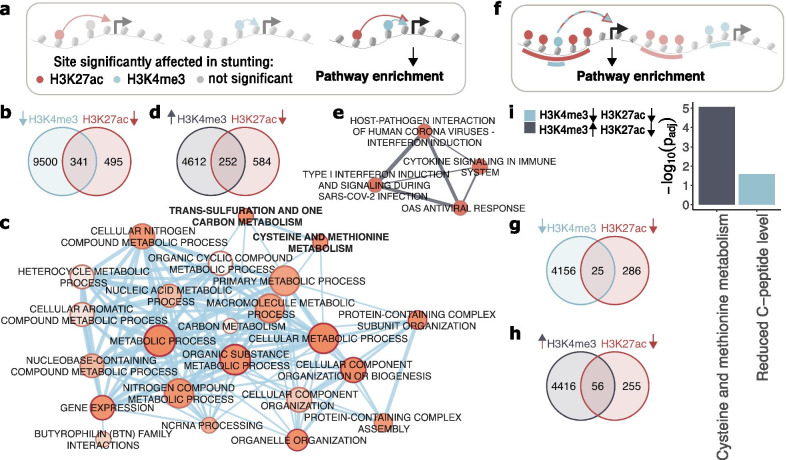
Integrative analysis of H3K27ac and H3K4me3 in 1-year-old stunted children. **a** Overview of the “gene-centric” approach in which each differential H3K27ac and H3K4me3 region was assigned to its target gene(s). Only genes predicted to be regulated by both H3K27ac and H3K4me3 were functionally annotated. **b**, **d** Venn diagrams show overlap of genes regulated by H3K27ac regions that were downregulated in stunting (red circle), and H3K4me3 regions that were downregulated (blue circle) and upregulated (grey circle) in stunting respectively. **c**, **e** Networks of biological terms as in Fig. 3b generated for overlapping genes from **b**, and **d** respectively. The color code of network edges corresponds to the color code of H3K4me3 circles in the associated Venn diagrams. **f** Overview of the “region-centric” approach. Significantly affected H3K27ac regions that overlap significantly affected H3K4me3 regions were assigned to target genes, which were functionally annotated. **g**, **h** Venn diagrams showing intersections of significantly downregulated H3K27ac regions (red circle) in stunting with significantly downregulated (blue circle) or upregulated (grey circle) H3K4me3 regions in stunting. **i** Pathway enrichment of genes associated with overlapping regions from **g**, **h**. Bars are color-coded accordingly with H3K4me3 circles in Venn diagrams

First, we took a gene-centric approach in which significantly affected H3K27ac and H3K4me3 regions were associated with target genes and pathway enrichment analysis was then performed on those genes present in both lists (Fig. 5a). We separately analyzed genes whose H3K4me3 and H3K27ac regions were both downregulated in stunting (341 genes in Fig. 5b), and genes whose H3K4me3 regions were upregulated, but H3K27ac downregulated in stunting (252 genes in Fig. 5d). Functional enrichment of genes for which both marks were downregulated is shown in Fig. 5c, and for genes with upregulated H3K4me3 but downregulated H3K27ac in Fig. 5e. The results in Fig. 5c show predominantly biological terms connected to metabolism and cellular organization and thus suggest overall reduced metabolic capacity and possible metabolic rewiring in stunted children. Interestingly, one-carbon metabolism was again detected, providing further support for the hypothesis that stunted children suffer from aberrant one-carbon metabolism. The list from Fig. 5b contains the LDL receptor related protein 1 (LRP1) gene, whose role in stunting was previously described by us and supported in a mouse model [17]. The results in Fig. 5e show terms associated with viral infection, which may suggest that the loss of H3K27ac is at least partially compensated for by gain of H3K4me3 to maintain immunological responses at the transcriptional level to some extent.

Next we investigated the relationship between H3K27ac and H3K4me3 at 1 year of age using a region-centric approach in which significantly affected H3K27ac regions that overlap with significantly affected H3K4me3 regions were associated with genes and these gene sets were investigated for functional enrichment (Fig. 5f). Overall, there was no relationship between the log_2_(fold-changes) in H3K27ac and H3K4me3 overlapping regions (Additional file 2: Fig. S9b, c). The concordant overlapping regions that were both decreased in stunting were associated with 25 genes (Fig. 5g), and the discordant regions where stunted children lost H3K27ac but gained H3K4me3 were associated with 56 genes (Fig. 5h). The functional enrichment results for both gene sets are shown in Fig. 5i. The concordant regions were enriched in the C-peptide level pathway. This pathway is associated with low insulin production and is an indicator of disruptions in glucose metabolism leading to diabetes, a disease that tends to emerge in stunted individuals [8, 9] (Fig. 1). Discordant regions were found to be enriched in cysteine and methionine metabolism pathways. Importantly, the pattern of H3K4me3 redistribution observed in stunting is similar to changes that result from methionine restriction [17] and both are associated with non-random effects on specific metabolic genes. Collectively, these results provide yet another argument in support of the conclusion that stunting is associated with altered one-carbon metabolism.

Lastly, we restricted the functional annotation to strictly overlapping H3K27ac and H3K4me3 regions (rather than annotating whole H3K27ac regions that overlap with H3K4me3, Additional file 2: Fig. S9d). The pathway enrichment analysis results of the associated genes are presented in Additional file 2: Fig. S9e. Here we observed the association of downregulated H3K4me3 and H3K27ac regions with NOTCH signaling genes, a pathway crucial in development, whereas H3K4me3 regions upregulated within H3K27ac regions show association with genes involved in the acute inflammatory response as well as vitamin B12 metabolism, a component of one-carbon metabolism with a previously reported association with stunting [30].

## Discussion

**Fig. 6:**
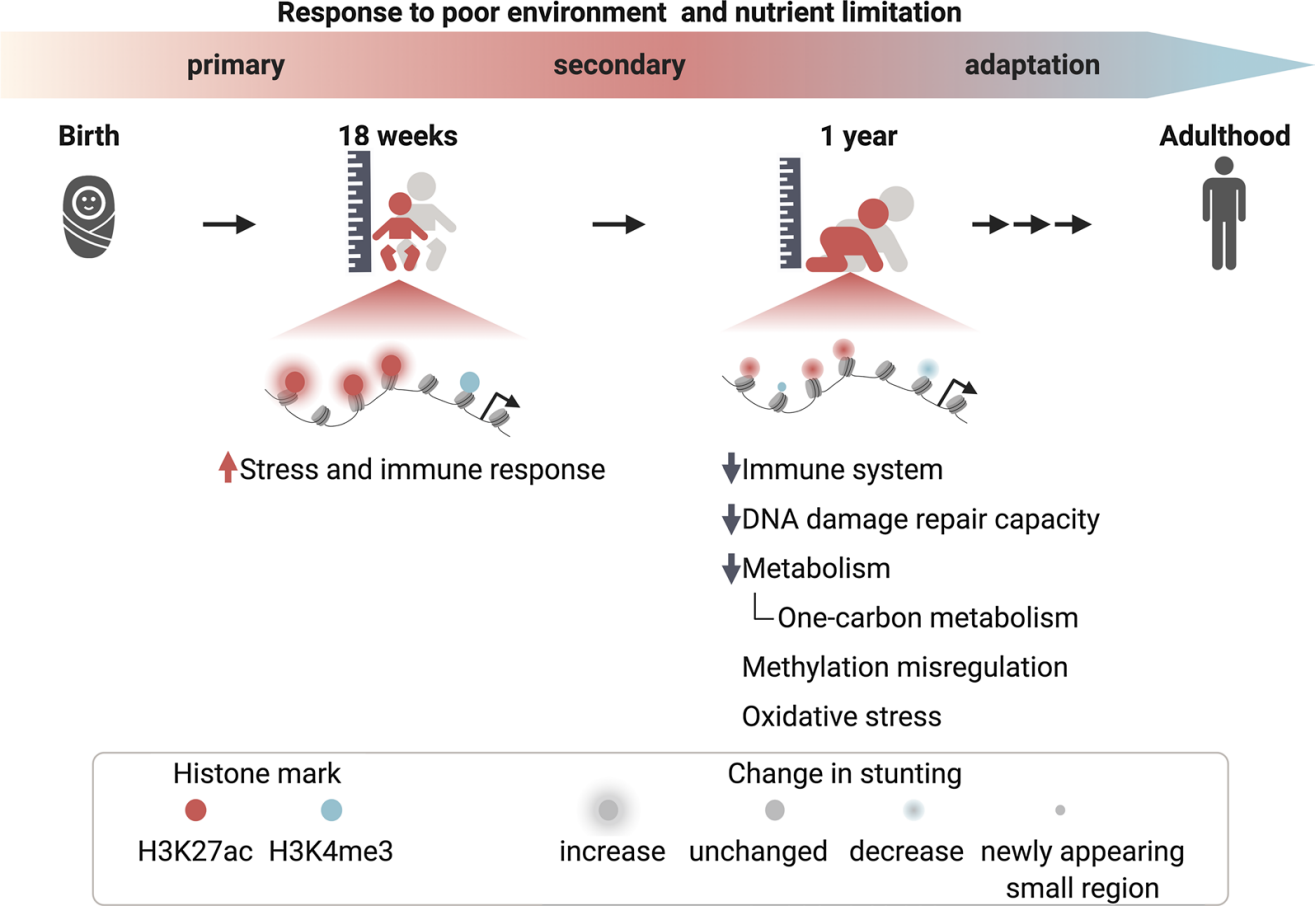
Working model depicting changes to the epigenetic landscape in stunting. The model highlights changes to H3K27ac and H3K4me3 landscapes in 18-week-old and 1-year-old children as they become more severely stunted, along with biological terms associated with these changes

The results presented here and in our previous work are summarized in the model in Fig. 6. While we did not observe any significant changes in the H3K4me3 profiles of 18-week-old children in association with the emergence of stunting, increased levels of H3K27ac were functionally linked to activation of stress and immune response pathways. Importantly, this occurred before the overt appearance of the stunted phenotype in these children. It has been reported previously that H3K27ac is one of the most dynamic histone marks during immune cell reprogramming [31, 32], which could contribute to why changes were observed in H3K27ac but not H3K4me3. Taken together, we suggest that differential enhancer activation in early infancy represents a response to initial encounters with a poor environment, including an increased pathogen load and nutritional challenges.

It is metabolically expensive to maintain immune responses under prolonged periods of increased pathogen exposure [33], and likely unsustainable for growing infants, especially under conditions of nutrient limitation [34]. This is possibly one of the reasons for the epigenetic changes we observed in 1-year-old stunted children, which we hypothesize occur as secondary responses to a poor environment and nutrient limitation (Fig. 6). These secondary epigenetic responses consist of global H3K27ac downregulation and a pattern in which H3K4me3 was redistributed from its canonical sites close to TSSs to distal ectopic loci. Functional annotation of both marks indicates that the immune system of stunted children is compromised, which has been well established on a functional level [6, 35]. The directionality of the epigenetic changes further suggests that immune cells of stunted children become functionally inactive, i.e. exhausted or immune-paralyzed [6, 28]. Immune system dysfunction is tightly linked to DNA damage and to alterations of DNA damage pathways [36] which are responses to elevated reactive oxygen species (ROS), and oxidative stress [37], all of which appear to be affected in 1-year-old stunted children according to our results (Fig. 4b). Furthermore, aberrant DNA-repair and associated pathways were linked with multiple deficiencies including growth retardation and cognitive impairment [22], which are features of the stunted phenotype.

One of our long-term goals is to identify nutritional interventions that address the specific needs of this stunted child population. The changes we detected in H3K27ac in 1-year-old children suggest downregulation of one-carbon metabolic pathways (Fig. 4b, c). This is particularly interesting since our previously published H3K4me3 results uncovered a similarity between the pattern in stunted children and the pattern in methionine-starved cells [38]. We further provide evidence through H3K27ac-H3K4me3 integrative analyses that changes in one-carbon metabolism play an important role in stunting, as both marks are altered at genes in this pathway in stunted children (Fig. 5c, i). Methionine is an essential amino acid and a precursor for generating the methyl group donor, SAM. High-quality protein is metabolically expensive, and a plausible hypothesis is that stunted children have altered levels of one-carbon metabolites as a result of inadequate methionine consumption [39]. Such an inadequacy could be addressed through increased availability of complete protein or methionine supplementation in the diet. The consequences of reduced methionine levels on DNA methylation have been previously discussed in acute undernutrition [40]. The roles of one-carbon metabolite levels on DNA methylation during pregnancy as well as on fetal growth outcome have also been studied [41–43]. While low levels of one-carbon metabolites have been previously identified in undernourished children in low-income countries and its supplementation showed promising results [44–46], these studies have focused on levels of one-carbon substrates (folate, choline and betaine) or one-carbon cofactors (vitamins B_2_, B_6_ and B_12_). In contrast, here we identify probable downstream gene targets associated with altered one-carbon metabolism in this population.

Intriguingly, most of the genes highlighted in Fig. 4c, which are associated with significant loss of H3K27ac in stunting were also identified in the list of genes with loss of H3K4me3 in stunting. The distribution of the affected genes within the pathway (Fig. 4c) may suggest (i) methionine deficiency as discussed above, (ii) changes in glycolytic flux, and with it associated (iii) impairment in serine production. Alterations in glycolysis and serine biosynthesis have been associated with cognitive impairment [47], a common health issue in stunting. Furthermore (ii) and (iii) have been previously linked with some of the modules shown in Fig. 4b. For example, serine levels have been shown to impact senescence (note the oxidative stress-induced senescence term in Fig. 4b) [48], and enzymes in the glycolytic pathway such as lactate dehydrogenase A (LDHA) have been shown to be important in immune cell activation [49]. Intriguingly, the biological responses associated with these metabolites and those that are also observed in stunted children have been shown to be at least partially reversed by metabolite supplementation [38, 48, 50].

Although our ChIP-seq data were obtained from PBMCs, we see little evidence that the overall pattern changes reported here are attributable to changes in a particular cell subtype within these samples. The data were analyzed in two complementary ways: (i) we mapped the normalized ChIP-seq signal from individual samples onto cell type-specific enhancers defined by Andersson et al. [21] (Additional file 2: Fig. S5), and (ii) we mapped the normalized cell type-specific chromatin accessibility signals onto significantly affected regulatory elements (Additional file 2: Fig. S7). The results indicate that these changes are not cell type-specific, a conclusion supported by our prior results [17]. A possible exception is shown in Additional file 2: Fig. S7b, which indicates that differentially affected H3K27ac regions in 1-year-old children show slightly higher enrichment in open chromatin regions of monocytes and dendritic cells compared to other subpopulations. These findings are consistent with results from a cohort of stunted infants in Zimbabwe, which showed a decline of the CD14^+^ monocyte population in 1-year-old stunted children [51], as well as results from an animal model of protein malnutrition in which protein-deficiency led to dysfunctional dendritic cells and non-responsiveness to vaccination [50].

## Conclusions

We analyzed H3K27ac, a histone mark associated with active enhancers in PBMCs of healthy and stunted infants aged 18 weeks and 1 year. We identified sites with increased H3K27ac levels in 18-week-old children destined to become stunted, and conversely decreased H3K27ac levels in 1-year-old children who became stunted. Functional analyses associated these sites primarily with immune system and stress response genes. In addition, the data from 1-year-old children point to general changes in metabolic capacity and specifically suggest alterations in one-carbon metabolism that we hypothesize may result from nutritional reduced levels of one-carbon precursors.

## Methods

### Human peripheral blood mononuclear cells (PBMC) samples

Deidentified PBMC samples from 18-week-old (*n* = 14) and 1-year-old (*n* = 22) children enrolled in the PROVIDE study (*n* = 700) [19] were obtained in collaboration with icddr,b (International Centre for Diarrhoeal Disease Research, Bangladesh) in Dhaka, Bangladesh. The study was approved by the Ethical Review Board of icddr,b (FWA 00001468) and the Institutional Review Boards of the University of Virginia (FWA 00006183) and the University of Vermont (FWA 00000727). Within 7 d after giving birth, screening for eligibility and study consenting occurred in the household by trained Field Research Assistants. Informed consent was obtained for all participating children (trial registration: ClinicalTrials.gov NCT01375647).

### H3K27ac ChIP-seq

ChIP-seq experiments were performed as previously described in detail in Uchiyama et al. [17]. In brief, PBMC samples from 18-week-old and 1-year-old children were fixed with formaldehyde, chromatin isolated and sheared, 0.02% (microgram/microgram) *Drosophila* chromatin (cat #53083, Active Motif, Carlsbad, CA, USA) was added for spike-in normalization [52]. DNA fragments were isolated by immunoprecipitation with H3K27ac antibodies (12.5 μl per 100 μg chromatin protein solution, cat# C15410-196, lot# A1723-0041D, Diagenode, Denville, NJ, USA), and sequencing libraries constructed using the Illumina TruSeq ChIP Library Preparation Kit following the manufacturer instructions. Libraries were sequenced using an Illumina NextSeq500 instrument with high capacity cartridge in the University of Virginia DNA Sciences Core Facility, yielding between 41 and 72 million 150 bp single-end reads per sample.

### ChIP-seq data preprocessing

Datasets were generated from samples from 18-week-old (14 samples) and 1-year-old (22 samples) children, including both males and females and with a broad spectrum of HAZ and ΔHAZ scores (Fig. 2a, Additional file 2: Fig. S1a–c, j–l) that represent the distribution of HAZ and ΔHAZ scores of children enrolled in the PROVIDE study (Additional file 2: Fig. S2a–e). Raw H3K27ac sequences were aligned to the human genome (version hg19) with bowtie2 (2.2.6.) [53] using default settings. The resulting files were processed to remove unmapped reads and then converted to BAM format using Samtools (0.1.19-4428 cd) [54]. ENCODE-defined blacklisted sites [55] were removed using bedtools (v2.26.0) *intersect* [56]. Quality of each dataset was assessed with FastQC (v0.11.5) [57] in combination with MultiQC (1.2) [58]. Peaks identifying H3K27ac enriched regions were called using MACS2 (2.1.1.20160309) [59] against input with parameters set to—broad—broad-cutoff 0.01. Separately identified peaks with MACS2 settings—broad—broad-cutoff 0.05 were stitched together into putative enhancers and superenhancers with ROSE [60, 61] with recommended parameters -s 12,500 -t 2500. Based on visual inspection of peaks called only with MACS2 and of enhancers called with ROSE in Integrated Genomics Viewer (IGV) [62], enhancers called with ROSE were used for construction of count tables used in downstream differential analysis. To create the count table BED files with enhancer coordinates were merged into a single BED file using bedtools *merge* with default parameters, this was done for the two age groups separately, providing separate sets of putative enhancers for 18-week-old and 1-year-old children. Coverage of individual samples over the final region sets was then enumerated with bedtools *multicov* with default settings, providing separate count tables for each age group, which were then used as an input for differential analysis described in further section of methods. Count tables were also created in the same way for superenhancers.

Sequencing reads were also mapped to the *Drosophila* dm6 genome to obtain read counts for spike-in normalization factors. All necessary hg19 and dm6 files including genome indexes, chromosome sizes, etc., were obtained with refgenie (0.9.3) [63].

To visually inspect individual datasets and create genome browser snapshots, normalized bigWig files were created from BAM files using bedtoos *genomcov* with argument-scale set to a normalization factor for a given sample (see section on data normalization below about calculation of normalization factors) followed by use wigToBigWig tool [64].

### Data normalization

Prior to differential analysis, it is essential to correctly normalize count tables. *Drosophila* spike-in chromatin was used in our experimental set up to correct for global changes (Additional file 2: Fig. S3a). If the ratio of human to *Drosophila* chromatin stays the same, then the percentage of reads mapped to *Drosophila* genome out of all sequencing reads, should be different only if there is a global gain or loss of a histone mark under certain conditions (e.g. in stunted children), as shown in illustration in Additional file 2: Fig. S3b, c. In the case that there is no global change between conditions, the percentage of recovered *Drosophila* sequences should be roughly the same (Additional file 2: Fig. S3c). The percentage of reads mapped to *Drosophila* should not, however, be dependent on any other factor, like sequencing depth, which is something that was observed in the data presented here (Additional file 2: Fig. S3d). To account for this relationship between the number of sequencing reads and the percentage of reads mapped to *Drosophila*, we regressed out the linear relationship between these two variables and recalculated the normalization factors based on residuals recovered from the linear model. The corrected number of reads mapped to *Drosophila* was then calculated as:

$${n}_{\mathrm{dm}}=\frac{\left(\mathrm{residual}+1\right)\cdot n}{100} ,$$

where *n*_dn_ is the corrected number of reads mapped to *Drosophila*, *residual* is a linear model residual corrected by adding 1 to eliminate negative numbers, and *n* is the total number of sequencing reads for a given sample. The final normalization factors were then obtained by dividing *n*_dm_ with an arbitrary constant, here the constant was chosen to be 500,000. The final values (Additional file 3: Table S2) were used for count table normalization and bigWig file scaling.

### Differential analysis

Differential analysis using normalized count tables was carried out by DESeq2 (1.30.0) [65] with design set to sex + ΔHAZ (18 wk) for 18-week-old children and sex + ΔHAZ (1 yr) for 1-year-old children. This allowed for identification of regions for which the coverage changed dynamically with increasing ΔHAZ score, while controlling for sex differences. Significantly affected regions were then defined as those with an FDR-corrected *p* value < 0.05.

The significance of the apparent global shifts in acetylation in stunted children was assessed by performing one-sample two-sided Student’s *t*-test on log_2_(fold-changes) with *μ* = 0. Results with *p* < 0.05 were considered significant.

The alluvial plot assessing changes that occurred in stunted children between 18 and 52 weeks was created by first finding overlaps between regions from the two count tables (18 weeks, 1 year) with bedtools *intersect* function with argument -wao, which identifies overlapping regions, but instead of returning their intersection, the overlapping regions are returned in their original form. This allows connecting results from differential analysis to the regions. Based on log_2_(fold-changes), regions were classified as “up” or “down” in stunting for each group or “not present” if a region in one age group does not overlap with any of the regions defined in the other age group. Data in this form was then plotted as an alluvial plot with ggalluvial (0.12.2) R package [66]. Log_2_(fold-changes) connected to overlapping regions (not regions present only in one age group) were also plotted as a scatter plot and their correlation was assessed by calculating Pearson’s correlation coefficient. Regions upregulated at 18-weeks and downregulated at 1-year in stunting selected for functional annotation were selected based on the weighted difference in log_2_(fold-change) at a given age weighted by FDR-corrected *p* values:

$$\begin{aligned} {\text{weighted}}\;{\text{FC}} & = \left[ {\log_{2} \left( {\text{fold-change}} \right)_{{1\;{\text{yr}}}} - \log_{2} \left( {\text{fold-change}} \right)_{{18\;{\text{wk}}}} } \right] \\ & \quad \cdot \left[ { - \log_{10} \left( {p_{{{\text{adj}}}} } \right)_{{18\;{\text{wk}}}} + \left( { - \log_{10} \left( {p_{{{\text{adj}}}} } \right)_{{1\;{\text{yr}}}} } \right)} \right]. \\ \end{aligned}$$

Intersections of the top 10% of these enhancers were functionally annotated as described below.

Principal component analysis (PCA) was carried out as described in the DESeq2 vignette using regularized logarithm transformation; all regions were used for analysis.

### Gene annotation and functional annotation

H3K27ac regions of interest were associated with genes using the EnhancerAtlas 2.0 database [67]. A database for PBMCs was created by downloading EnahncerAtlas2 defined enhancer-gene interactions for PBMC-relevant cell types, namely CD4+, CD8+, CD14+, CD19+, CD20+, GM10847, GM12878, GM12891, GM12892, GM18505, GM18526, GM18951, GM19099, GM19193, GM19238, GM19239, GM19240, PBMC cells. Individual text files were reformatted into tab delimited files, in which the first three columns consisted of the region coordinates (chr, start, end) followed by columns with gene identifiers, and cell type. The reformatted files were then concatenated and sorted, giving rise to the final custom PBMC EnhancerAtlas 2.0 database. Regions of interest were assigned to genes by finding overlaps with the regions in the created database.

Functional enrichment analysis using gene lists of interest was performed as recommended by Reimand et al. [68]. Specifically, gene lists were functionally annotated by g:Profiler [69] with data sources restricted to GO biological processes, KEGG, Reactome, and WikiPathways, otherwise with default settings. Outputs from g:Profiler were visually represented in two ways: (1) shorter lists (number of terms ≤ 10) were visualized based on information in CSV files as bar graphs with bar height set to − log_10_(*p*_adj_), (2) longer lists (number of terms > 10) were visualized as networks using EnrichmentMap (3.3) [70] Cytoscape (3.8.0) [71] application. EnrichmentMap requires the output from g:Profiler in the form of a GEM file along with a GMT file providing information about which gene belongs to a given biological term. GMT files for GO biological processes, Reactome, and WikiPathways were obtained from g:Profiler, and combined with GMT file for KEGG, that was obtained separately from EnrichmentMap website (https://enrichmentmap.readthedocs.io/en/latest/GeneSets.html). The nodes in resulting networks represent individual biological terms, node size correspond to the term size, node color to the significance of the term, and edges were formed based on the number of overlapping genes between the two connected terms. Biological terms were then manually divided into clusters based on their similarity. Due to the large complexity of results obtained in functional annotation of genes associated with regions that changed between 18 weeks and 1 year in stunting, GO biological processes were eliminated in this analysis.

Genes involved in the response to viral infection in data from 18-week-old children were validated for functional enrichment with STRING [72] with text mining off.

Enrichment analysis was also performed for regions of interest with LOLA [73] using CISTROME [74, 75] databases of transcription factors (termed here as DNA-binding factors) and histone marks filtered for blood cells. The CISTROME database was available only for the hg38 genome assembly, so H3K27ac regions of interest were first converted to hg38 annotation with liftOver [76]. Significantly affected regions defined by DESeq2 were used as “userSet”, and all regions from a count table as “userUniverse.” Significant results were identified as those with *q*-value < 0.05, and presented in form of a dot plot where the *x*-axis represents different cell types, the *y*-axis represents different histone marks or DNA-binding factors, and the size along with the color of a dot reflects the most significant *q*-value for a given cell type—histone mark or cell type—DNA-binding factor combination. Due to the very long list of significant DNA-binding factors, only the top quartile of the results ranked by *q*-value was visualized.

### Cell-type specificity analysis

Two approaches were employed to identify cell-specific responses from the batch ChIP-seq experiments. In the first approach, average normalized H3K27ac profiles were generated over enhancer regions specific for a given cell type (these include B-cells, T-cells, dendritic cells, macrophages, monocytes, and natural killer cells) defined by Andersson et al. [21], as shown in Additional file 2: Fig. S5a. To use truly cell specific regions, any intersecting regions among the different cell types were excluded from the analysis. Average profiles were obtained by mapping normalized bigWig files onto the cell specific BED files with deepTools (3.3.1) [77] *computeMatrix* function with arguments –referencePoint center -b 1000 -a 1000 followed by *plotProfile* function. The average profiles were plotted in R with ggplot2 (3.3.2) with smoothing parameters set to geom_smooth(span = 0.2).

The GenomicDistributions package (0.99.4) [78] was used as a complementary approach, where normalized open chromatin signal from ENCODE DNase-seq and ATAC-seq experiments across different cell types was mapped onto the H3K27ac regions of interest (Additional file 2: Fig. S7), i.e. onto significant regions identified with DESeq2.

### H3K4me3 data

H3K4me3 datasets from 1-year-old children from Uchiyama et al. [17] were pre-processed to generate count tables as described in detail in Uchiyama et al. [17]. Differential analysis was performed by DESeq2 (1.30.0) with default normalization and design set to sex + ΔHAZ (1 yr). Significant peaks that were down in stunted children were previously shown to be closer to transcription start sites (TSSs), and were therefore associated with genes by using GREAT [79], while significant peaks that are upregulated in stunted children tended to be further from TSSs and enriched in enhancer regions [17], and were therefore associated with genes by EnhacerAtlas2-like H3K27ac regions. Venn diagrams for co-regulated genes by H3K4me3 and H3K27ac or for the intersections of the regions were created with VennDiagram package (1.6.20) [80]. Pathway enrichment analysis of coregulated genes was performed as described for H3K27ac datasets. Correlation of log_2_(fold-changes) of intersecting H3K4me3 and H3K27ac regions was evaluated by calculating Pearson’s correlation coefficient.

Illustrations were generated with BioRender [81]. Other R packages used in the analysis for data wrangling and plot editing: tidyverse (1.3.0) [82], GenomicRanges (1.42.0) [83], Hmisc (4.4-2) [84], ggpubr (0.4.0) [85], ggmosaic (0.2.0) [86], ggrastr (0.2.1) [87].

## Supplementary Information

**Additional file 1: Table S1.** List of samples and associated metadata.

**Additional file 2.** Supplementary figures (S1–S9).

**Additional file 3: Table S2.** Pre-processing results.

**Additional file 4: Table S3.** H3K27ac differential analysis results for 18-week-old children.

**Additional file 5: Table S4.** H3K27ac differential analysis results for 1-year-old children.

**Additional file 6: Table S5.** Significant histone marks identified with LOLA for 18-week-old children.

**Additional file 7: Table S6.** Significant DNA-binding factors identified with LOLA for 18-week-old children.

**Additional file 8: Table S7.** Significant histone marks identified with LOLA for 1-year-old children.

**Additional file 9: Table S8.** Significant DNA-binding factors identified with LOLA for 1-year-old children.

**Additional file 10: Table S9.** H3K4me3 differential analysis results for 1-year-old children.

## Data Availability

The datasets supporting the conclusions of this article are available in dbGaP repository, phs001073.v2.p1, https://www.ncbi.nlm.nih.gov/gap/. DbGaP identifiers are listed in Additional file 1: Table S1. Important scripts associated with this manuscript are available from: https://github.com/AubleLab/H3K27ac_in_stunting (https://zenodo.org/badge/latestdoi/339230344).

## Abbreviations

ChIP-seq: Chromatin immunoprecipitation followed by sequencing
CVD: Cardiovascular diseases
FDR: False discovery rate
H2AK119: Histone H2A mono-ubiquitination on lysine 119
H3K4me2: Histone H3 dimethylation on lysine 4
H3K4me3: Histone H3 trimethylation on lysine 4
H3K27ac: Histone H3 acetylation on lysine 27
H3K27me3: Histone H3 trimethylation on lysine 27
HAZ: Height-for-age *z*-score
ΔHAZ (18 wk): Difference in height-for-age *z*-scores between birth and 18 weeks of age
ΔHAZ (1 yr): Difference in height-for-age *z*-scores between birth and 1 year of age
JMJD1C: Jumonji domain containing 1C
LDHA: Lactate dehydrogenase A
LRP1: LDL receptor related protein 1
PBMC: Peripheral blood mononuclear cells
PCA: Principal component analysis
PPP: Pentose phosphate pathway
PROVIDE: Performance of rotavirus and oral polio vaccines in developing countries
ROS: Reactive oxygen species
SAM: S-adenosyl methionine
SSP: Serine synthesis pathway
T2D: Type 2 diabetes
TC-NER: Transcription coupled nucleotide excision repair
TSS: Transcription start site
WHO: World Health Organization
